# Nano-Sized Extracellular Matrix Particles Lead to Therapeutic Improvement for Cutaneous Wound and Hindlimb Ischemia

**DOI:** 10.3390/ijms222413265

**Published:** 2021-12-09

**Authors:** Sang Su Ha, Jung-Hyun Kim, Cininta Savitri, Donghoon Choi, Kwideok Park

**Affiliations:** 1 Center for Biomaterials, Korea Institute of Science and Technology (KIST), Seoul 02792, Korea; 024730@kist.re.kr (S.S.H.); casavitri@kist.re.kr (C.S.); 2 Division of Cardiology, Department of Internal Medicine, Severance Cardiovascular Hospital, Yonsei University College of Medicine, Seoul 03722, Korea; jhkim915@yuhs.ac; 3 Division of Bio-Medical Science and Technology, KIST School, University of Science and Technology (UST), Seoul 02792, Korea

**Keywords:** fibroblast-derived matrix (FDM), decellularization, suspended FDM, Pluronic F127, hyaluronic acid, wound healing, hindlimb ischemia

## Abstract

Cell-derived matrix (CDM) has proven its therapeutic potential and been utilized as a promising resource in tissue regeneration. In this study, we prepared a human fibroblast-derived matrix (FDM) by decellularization of in vitro cultured cells and transformed the FDM into a nano-sized suspended formulation (sFDM) using ultrasonication. The sFDM was then homogeneously mixed with Pluronic F127 and hyaluronic acid (HA), to effectively administer sFDM into target sites. Both sFDM and sFDM containing hydrogel (PH/sFDM) were characterized via immunofluorescence, sol–gel transition, rheological analysis, and biochemical factors array. We found that PH/sFDM hydrogel has biocompatible, mechanically stable, injectable properties and can be easily administered into the external and internal target regions. sFDM itself holds diverse bioactive molecules. Interestingly, sFDM-containing serum-free media helped maintain the metabolic activity of endothelial cells significantly better than those in serum-free condition. PH/sFDM also promoted vascular endothelial growth factor (VEGF) secretion from monocytes in vitro. Moreover, when we evaluated therapeutic effects of PH/sFDM via the murine full-thickness skin wound model, regenerative potential of PH/sFDM was supported by epidermal thickness, significantly more neovessel formation, and enhanced mature collagen deposition. The hindlimb ischemia model also found some therapeutic improvements, as assessed by accelerated blood reperfusion and substantially diminished necrosis and fibrosis in the gastrocnemius and tibialis muscles. Together, based on sFDM holding a strong therapeutic potential, our engineered hydrogel (PH/sFDM) should be a promising candidate in tissue engineering and regenerative medicine.

## 1. Introduction

Blood supply is a crucial event in the body to transport oxygen, nutrients, and cells for maintaining normal function of tissues and organs. Tissue regeneration barely occurs without angiogenesis, which is the formation of new vessels from the pre-existing blood vessels and involves endothelial sprouting and splitting of vessel lumens [1,2]. Since insufficient blood supply is a major cause of several chronic diseases, numerous studies have concentrated on the technologies for advanced angiogenesis by using various strategies, such as scaffolds, hydrogels, cells, growth factors, and/or chemokines [3,4,5].

Extracellular matrix (ECM), a highly organized complex of macromolecules and signaling factors, is a promising biomaterial, while ECM directly influences cell adhesion, proliferation, migration, and differentiation [6,7]. Due to its intrinsically biomimetic properties, decellularized ECM has been an attractive resource in tissue engineering that is mainly obtained from tissues, organs or even cells via decellularization processes [8]. Among them, our group has long investigated cell-derived ECM (CDM), as secreted and self-assembled by in vitro cultured cells. We have documented the biomimetic characteristics of CDM in terms of compositional diversity of natural ECM and bioactive growth factors/cytokines embedded in the CDM [9,10]. In fact, our previous studies demonstrated that CDM possesses a considerable capability of promoting angiogenesis in vitro and in vivo by providing a unique microenvironment for angiogenic cellular functions [11,12]. Technically it is possible to customize CDM by selecting proper cell type that is target tissue oriented. For practical use of CDM, it can also be incorporated with hydrogels and scaffolds due to its extremely soft property. To deliver sensitive biomolecules (growth factors, proteins, and exosomes), many research groups have combined them with biocompatible hydrogels [13,14,15].

In the present study, we prepared a human fibroblast-derived matrix (FDM) suspension (sFDM), in which decellularized FDM was processed into nano-sized FDM particles in a suspended state. As compared to FDM itself, one of the technical advances with sFDM is the highly improved homogeneity of FDM when we developed an FDM-based therapeutic formulation. To examine the therapeutic effect of sFDM in vivo, we utilized both Pluronic F127 and hyaluronic acid (HA) as an sFDM-delivery platform. Triblock copolymer Pluronic F127, composed of hydrophobic poly (propylene oxide) (PPO) and hydrophilic poly (ethylene oxide) (PEO), is a typical example of an injectable, thermo-sensitive hydrogel [16,17,18]. Pluronic F127 is then physically mixed with HA to improve the overall mechanical strength of the final product. HA plays a crucial role in many cellular processes, including matrix assembly and angiogenesis [19,20,21]. A combination of both Pluronic F127 and HA produces a paste type, injectable hydrogel, and sFDM was subsequently incorporated into it. We hypothesize that such hydrogel/sFDM coupling may realize distinct benefits: manufacturing of sFDM-based therapeutic formulation, effective delivery of sFDM into target sites, and its therapeutic effectiveness in vivo. sFDM as a major therapeutic ingredient contains several angiogenic factors and thus should play an active role in enhancing angiogenesis and wound healing. We examined therapeutic efficacy of sFDM-contained hydrogel via animal models of full-thickness wound and hindlimb ischemia.

## 2. Results

### 2.1. Preparation and Characterizations of sFDM and PH/sFDM

**Figure 1:**
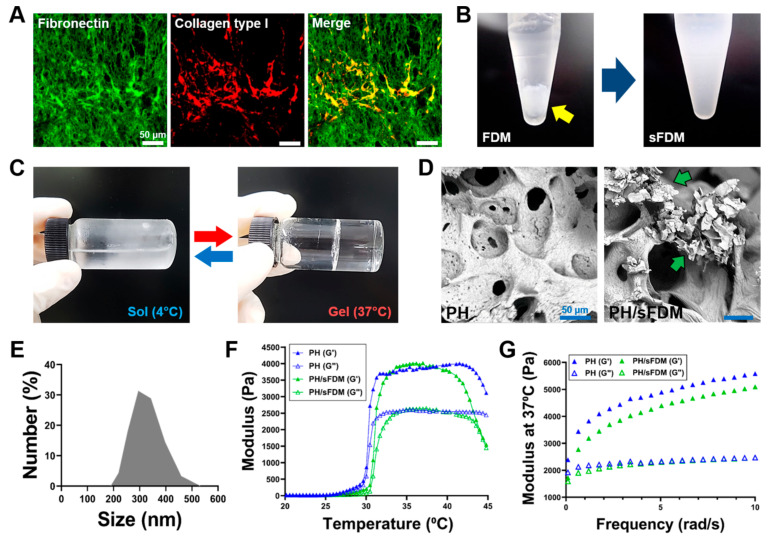
Preparation of suspended fibroblast-derived matrix (sFDM) and hydrogels and their characterizations. (**A**) ECM proteins in the decellularized FDM via immunofluorescence staining: fibronectin (green) and collagen type I (red). (**B**) Appearance of FDM before and after (sFDM) ultrasonication. Yellow arrow shows the collected FDM from the culture plate. (**C**) Sol–gel transition behavior of the hydrogel (PH) at 4 and 37 °C. (**D**) Internal microstructure of PH and PH/sFDM hydrogels via SEM. Green arrows indicate sFDM particles. (**E**) Measurement of particle size distribution of sFDM. (**F**) Profile of thermosensitive gelation between 20 and 45 °C for both hydrogels (PH and PH/sFDM) taken at 6.28 rad/s. (**G**) Comparison of both storage (G’) and loss (G”) moduli between the two hydrogels at body temperature.

Once human FDM was prepared by the decellularization process, the representative ECM proteins (fibronectin and collagen) in the FDM were found richly and evenly distributed via immunofluorescence (Figure 1A). DAPI staining confirms the successful clearance of nucleic components (Figure S1). When the decellularized FDM on the culture plate was collected and transferred into the tube filled with deionized (DI) water, a homogenously suspended FDM (sFDM) dispersion was produced using ultrasonication (Figure 1B). Meanwhile, to effectively administer sFDM for therapeutic purpose, we also prepared a hydrogel system, in which both Pluronic F-127 and HA were physically mixed together. Such hydrogel showed a sol–gel transition behavior, where it was a solution at 4 °C but in a gel state at 37 °C (Figure 1C). The scanning electron microscopy (SEM) image of Pluronic/HA (PH) revealed a porous microstructure inside the hydrogel, whereas sFDM-containing PH (PH/sFDM) exhibited the sFDM particles embedded, without compromising the structural integrity of PH itself (Figure 1D). Examination of sFDM particle size distribution showed that most of the sFDM size was distributed in a range of 250 and 400 nm (Figure 1E). Rheological properties of the two hydrogels at the same applied frequency of 6.28 rad/s demonstrated that their thermosensitive behavior is quite similar to each other at the temperature range of 20–45 °C (Figure 1F). In addition, the measurement for viscoelastic property of hydrogels proved a slightly higher storage modulus (G’) of PH at 37 °C than that of PH/sFDM (Figure 1G).

### 2.2. Biological Properties of sFDM and PH/sFDM

**Figure 2:**
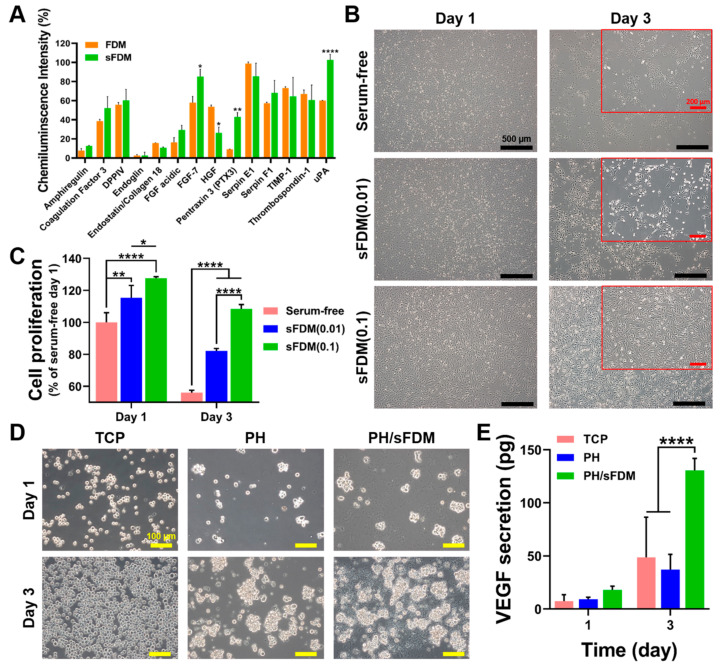
Examination of biological properties of sFDM in vitro. (**A**) Quantitative assessment of angiogenic factors profile of both FDM and sFDM. (**B**) Human umbilical vein endothelial cells (HUVEC) morphology and cell adhesion when treated with three different types of media on day 1 and 3. Red boxes show the enlarged images of cell morphology at 3 days. (**C**) Quantitative analysis of cell proliferation after three different treatments for up to 3 days as compared to that of serum-free group. (**D**) Cultivation of THP-1 cells on the TCP, PH, and PH/sFDM. (**E**) Determination of VEGF released from each group at specific time points (day 1 and 3). Statistically significant difference (* *p* < 0.05, ** *p* < 0.01, **** *p* < 0.0001).

Some bioactive factors included in the FDM and sFDM were separately analyzed using the angiogenic-related cytokines array kit (Figure 2A). We found that most of the angiogenic factors showed little difference between FDM and sFDM, while a few cytokines (FGF-7, HGF, PTX3, and uPA) exhibited statistically significant differences in chemiluminescence intensity. Meanwhile, regarding the effect of sFDM on cell behaviors, human umbilical vein endothelial cells (HUVECs) were cultivated under three different medium conditions (EBM-2, EBM-2 with 0.01 mg/mL sFDM, and EBM-2 with 0.1 mg/mL sFDM) for up to 3 days (Figure 2B). Cell morphology appeared normal on day 1 but cell detachment and dead cells were apparently noticed in the serum-free and sFDM (0.01) on day 3. Interestingly, only the sFDM (0.1) group showed good cell attachment. Quantitatively assessed, the proliferation of HUVECs on day 1 and 3 demonstrated that sFDM (0.1)-supplemented medium could maintain cell metabolic activity perfectly unlike the rest of the groups (Figure 2C). On the other hand, as an examination of immune response of hydrogels (PH and PH/sFDM), we grew THP-1 cells, a monocyte, on the hydrogels for up to 3 days (Figure 2D). Formation of cell clusters was apparently observed, especially on the PH and PH/sFDM. Quantitative analysis of the culture media revealed that a much higher amount of vascular endothelial growth factor (VEGF) was found in the PH/sFDM-derived medium compared to that of the TCP- or PH-derived one (Figure 2E). The difference of VEGF release level on day 3 was statistically significant compared to the other groups.

### 2.3. Hydrogel Administration to Murine Full-Thickness Skin Wound Model

**Figure 3:**
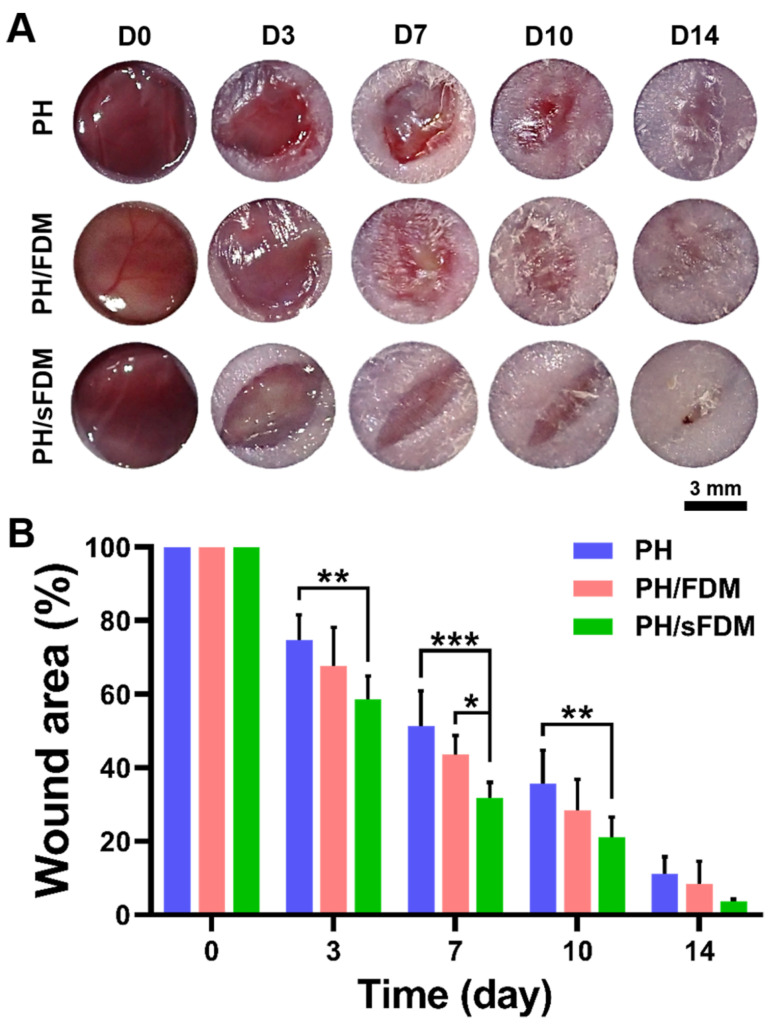
Observation of full-thickness wound closure with time. (**A**) Representative appearance of wounds treated with three test groups (PH, PH/FDM, PH/sFDM) on day 0, 3, 7, 10, and 14 post-surgery. (**B**) Quantitatively measured wound area ratio (%) at specific time points. Statistically significant difference (* *p* < 0.05, ** *p* < 0.01, *** *p* < 0.001).

To assess wound repair efficiency of hydrogels in vivo, we treated full-thickness wounds with three different types of hydrogels (PH, PH/FDM, and PH/sFDM), respectively and monitored the wound closure with time (Figure 3A). When the wound size at each time point (day 3, 7, 10, and 14) was quantitatively evaluated, PH/sFDM showed significantly better wound closure rate than PH hydrogel until 10 days (Figure 3B). There was no significant difference between FDM containing hydrogels (PH/FDM and PH/sFDM) except on day 7.

### 2.4. Histological Evaluation of Skin Wound Healing

**Figure 4:**
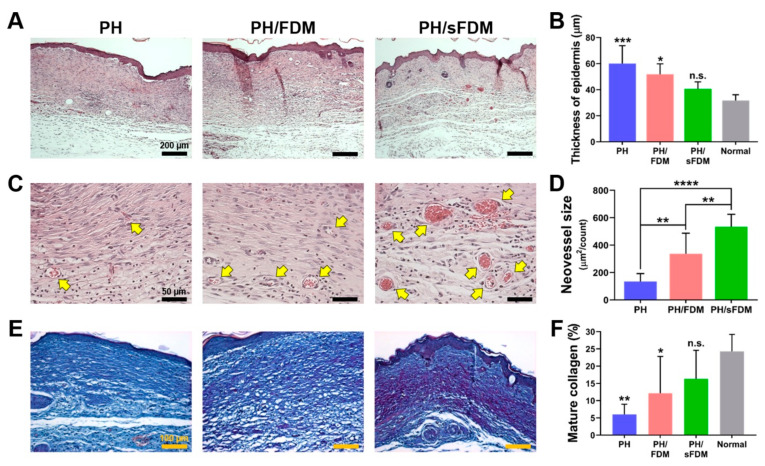
Histological analysis of the wound tissues on day 14. (**A**) Representative images of the regenerated skin tissues via hematoxylin and eosin (H&E) staining. (**B**) Measurement of epidermal thickness as statistically compared to that of normal (* *p* < 0.05, *** *p* < 0.001), n.s. means no significance. (**C**) High resolution images in the dermis. Yellow arrows indicate neovessels. (**D**) Quantitative evaluation of newly formed vessel size. Statistically significant difference (** *p* < 0.01, **** *p* < 0.0001). (**E**) Herovici staining of the regenerated tissues. Blue and purple color in the dermis represent young and mature collagen fibers, respectively. (**F**) Quantitative analysis of mature collagen deposition. Statistically significant difference compared to that of normal (* *p* < 0.05, ** *p* < 0.01).

The extent of wound healing in the regenerated skin tissue was examined at 14 days via hematoxylin and eosin (H&E) staining of the tissue sections (Figure 4A). Quantitatively measured, there was no significant difference of epidermal thickness between normal (31.7 ± 4.40 µm) and PH/sFDM (40.5 ± 5.43 µm) group but there was significant difference with PH (59.9 ± 13.83 µm) and PH/FDM (51.8 ± 8.01 µm) (Figure 4B). Newly formed blood vessels in the dermis were also confirmed by high-resolution images (Figure 4C). The PH/sFDM-treated wounds exhibited larger neovessels in comparison with PH or PH/FDM group. The neovessel size supported that PH/sFDM could develop much larger neovessels (543.3 ± 90.8 µm^2^) than the rest groups (134.5 ± 57.9 and 335.6 ± 150.6 µm^2^ for PH and PH/FDM, respectively) (Figure 4D). Moreover, when the regenerated collagen fibers in the dermis were subject to Herovici staining, which can distinguish young (blue) and mature (purple) collagen, the PH/sFDM group represented a relatively higher level of mature collagen deposition (Figure 4E). Quantitatively assessed, the percentage of mature collagen proved that the PH/sFDM-treated group (16.3 ± 8.27%) had no significant difference with normal skin (24.3 ± 4.89%) but this trend was not reproducible with the other test groups (6.0 ± 2.94 and 12.1 ± 10.68%, respectively) (Figure 4F). Current results demonstrate that sFDM-loaded hydrogel possesses a great wound healing potential.

### 2.5. Enhanced Blood Perfusion of Ischemic Hindlimb after Hydrogels Treatment

**Figure 5:**
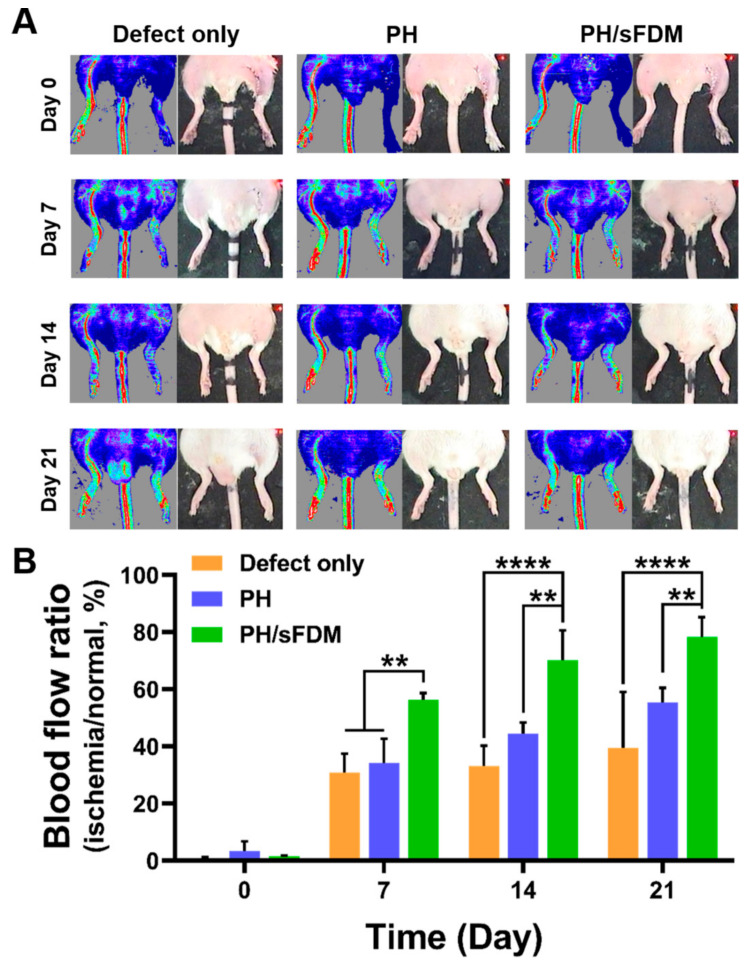
Blood perfusion recovery of ischemic hindlimb after the treatments for up to 21 days. (**A**) Representative images of laser Doppler perfusion imaging (LDPI) on day 0, 7, 14 and 21 post-treatments. Blue or dark-blue color indicates low and no perfusion, whereas red or yellow color indicates a high perfusion state. (**B**) Blood perfusion ratio was evaluated by comparing ischemic limb groups (defect only, PH, and PH/sFDM) with that of normal limb. Statistically significant difference (** *p* < 0.01, **** *p* < 0.0001).

For further assessment of therapeutic effects of PH/sFDM, we prepared ischemic hindlimb models and treated them using PH and PH/sFDM and observed laser Doppler perfusion imaging at 0, 7, 14, and 21 days post-surgery (Figure 5A). The blood perfusion was found to be completely blocked on day 0 in all the test groups. Improved blood perfusion was then observed at day 7 with the models treated with the defect group (PBS-treated), PH, and PH/sFDM, respectively. Quantitatively measured, blood perfusion of the PH/sFDM-treated group was 56.3 ± 2.36% compared to normal perfusion. However, the percentage was notably reduced to 34.2 ± 8.44% and 30.9 ± 6.45% for the PH and defect group, respectively (Figure 5B). We noticed on day 14 that while blood perfusion of PH/sFDM reached to 70.2 ± 10.29%, the one in PH and in defect remained at 44.4 ± 3.89% and 33.1 ± 7.15%, respectively. On day 21, blood perfusion was 78.2 ± 7.02% with PH/sFDM, 55.3 ± 5.17% with PH, and 39.3 ± 19.70% with PBS treatment. Indeed, the statistical difference was significant with time, especially at day 14 and 21 between PH/sFDM and defect or PH. Our results suggested that PH/sFDM could markedly improve blood perfusion in the ischemic hindlimb.

### 2.6. Attenuated Necrosis and Fibrosis in Ischemic Hindlimb

**Figure 6:**
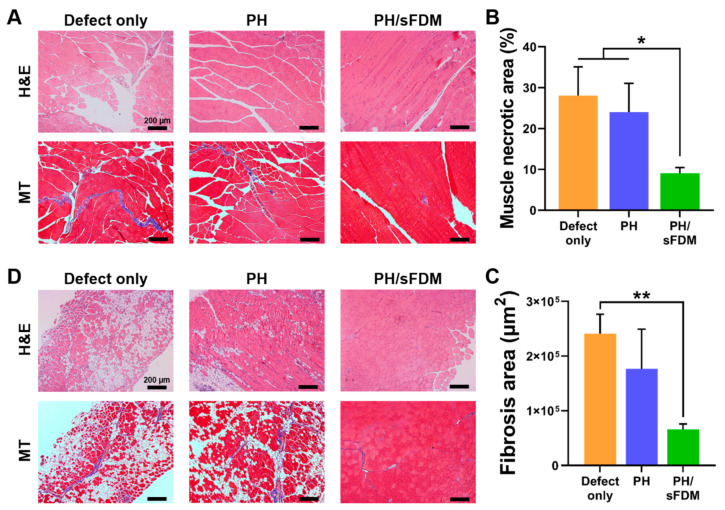
Histological analysis of ischemic hindlimb muscles at 21 days post-treatments. (**A**) Necrosis and fibrosis in the gastrocnemius muscles was examined using H&E and Masson’s trichrome (MT) staining, respectively. (**B**) Quantitative analysis of the necrotic area and (**C**) the fibrosis area as suggested in purple color. (**D**) Fibrosis area in the tibialis muscles was examined using H&E and MT staining. Statistically significant difference (* *p* < 0.05, ** *p* < 0.01).

For further analysis of ischemic hindlimb, we harvested ischemic gastrocnemius (Figure 6A) and tibialis (Figure 6D) muscle tissues at 21 days and analyzed them via H&E as well as Masson’s trichrome (MT) staining. The treatment with PH/sFDM seemed to have a protective effect against muscle degeneration, whereas severe necrotic lesions were observed with the defect group. The necrotic lesions were calculated as 9.04 ± 1.45% with PH/sFDM, but they were 24.0 ± 7.05 and 28.0 ± 7.08% with PH and defect, respectively (Figure 6B). In addition, fibrosis also decreased to a larger extent in the models treated with PH/sFDM. Quantitatively assessed, the area of fibrosis was 66,170 µm^2^ for PH/sFDM, 176,559 µm^2^ for PH, and 240,990 µm^2^ for defect (Figure 6C). The fibrosis area of PH/sFDM-treated group was considerably more reduced (3.5-fold) than the defect group. Moreover, when the tibialis muscles located in the lateral area of hindlimb were examined via H&E and MT staining, the results suggested that both muscle mass and morphology were maintained better than the other groups (Figure 6D).

## 3. Discussion

CDM is equipped with naturally derived ECM components, such as fibronectin, collagen, laminin, and proteoglycan, that are composed of natural microenvironments of organs and tissues. It also retains various signaling molecules that are crucial for cellular events, such as cell proliferation, migration, and differentiation. Unlike tissue or organ-derived ECM, CDM can be easily obtained from in vitro cultured cells and may be customized by selecting specific cell types for specific target tissue [6,7]. Therefore, CDM is considered as a promising, futuristic biomaterial in the field of regenerative medicine. Diverse cell culture methods for generating CDM exist: monolayer culture [22], embedding in degradable scaffolds [23], aggregated cell cluster [24], and cultivation on scaffold surface [25]. In this study, we prepared the FDM from monolayer cell culture via the mild decellularization process using non-ionic detergent, which can minimize ECM proteins damage.

Despite such advantages of CDM, there are some technical issues in handling CDM, one of them being CDM solubility. Most CDM components are proteins, and they have poor solubility in aqueous solution. If the FDM is supposed to be homogeneously mixed with hydrogel, preparation of the homogeneous form of FDM is a technically important point. Therefore, it was necessary to change FDM into a homogeneous state before combining with the hydrogels. For this purpose, we utilized an ultrasonicator and successfully produced nano-sized FDM particles in suspended state, as assessed via particle size distribution (Figure 1E). Due to ultrasonication being a decomposition method by physical force, we confirmed that the process hardly affected the biochemical factors of natural FDM, as assessed by angiogenesis antibody array (Figure 2A). Furthermore, we compared the wound healing effect using two hydrogels (PH/FDM and PH/sFDM) prepared via inclusion of two different forms of FDM, either sFDM or FDM. The results proved that skin tissue regeneration with homogeneously suspended FDM-containing hydrogel (PH/sFDM) was significantly better than that of a solid FDM-containing one (PH/FDM), as assessed via various parameters of wound healing (Figure 4). Regarding the sFDM concentration, current sFDM concentration (0.1%) in the hydrogels was determined via our preliminary in vivo test: two different sFDM concentrations (0.1 and 1%) were examined for wound healing effect and they showed no difference (Figure S2 in Supplementary Materials). We decided on 0.1% sFDM as an optimized sFDM amount and this reduced concentration undoubtedly results in some technical and economic benefit. As a delivery vehicle of sFDM, when Pluronic F127 was applied to skin wound, the hydrogel rapidly melted and flowed down. To overcome this problem, we tested HA and combined it with Pluronic F127 for reinforcing mechanical property of PH hydrogel, as previously reported [26]. HA also contribute to wound healing processes as reported in the matrix assembly and angiogenesis during tissue remodeling [27]. There was little difference between (PH and PH/sFDM) with respect to rheological properties, suggesting that sFDM does not significantly affect the gelation mechanism of PH hydrogel (Figure 1F,G).

The FDM has shown considerable potential in angiogenesis as documented by our previous studies [9,28]. FDM can be obtained from different cell sources, such as lung fibroblast or dermal fibroblast. We harnessed lung fibroblast instead of dermal for wound healing because our previous work has shown that the decellularized ECM from lung fibroblast has a relatively higher level of ECM proteins and angiogenic cytokines than dermal fibroblast [26]. However, since angiogenic capability of the sFDM has not been examined, we conducted in vitro biological assay before the in vivo test. To identify some angiogenesis related factors, we analyzed both FDM and sFDM using a sandwich-based angiogenesis antibody array kit (Figure 2A) and as a result, various cytokines were detected as follows: amphiregulin (AREG), coagulation factor Ⅲ (tissue factor), DPPIV (CD26), endoglin (CD105), endostatin (collagen XVIII), acidic fibroblast growth factor (FGF-1), fibroblast growth factor-7 (FGF-7), hepatocyte growth factors (HGF), pentraxin 3 (PTX3), serpin E1 (plasminogen activator inhibitor-1), serpin F1 (PEDF), tissue inhibitor of metalloproteinase (TIMP-1), thrombospondin-1 (TSP-1), and urokinase plasminogen activator (uPA). This result demonstrated that there was no major difference of bioactive factors before (FDM) and after (sFDM) the ultrasonication process, even though the intensity of some factors (FGF-7, PTX3, and uPA) significantly increased with sFDM. Together, our sonication method for generating sFDM barely affected the FDM’s biological factors.

As described above, sFDM contains diverse bioactive molecules as well as ECM components. Investigation of the sFDM effect on angiogenic and immune cells in vitro, showed that only the sFDM (0.1) group kept the shape of HUVECs, while serum-free and sFDM (0.01) led to cell detachment or death (Figure 2B). Interestingly, quantitative analysis of cell proliferation proved that sFDM (0.1) in serum-free conditions could help perfectly maintain cell metabolic activity (Figure 2C), indicating that the biomolecules and/or ECM compositions in sFDM may have a beneficial role in cell survival. Meanwhile, as an immune cell, monocytes have a critical role in new vessel formation by activating endothelial cells and vascular endothelial growth factor (VEGF); VEGF is associated with the initiation of angiogenesis and plays an important role in angiogenic function of endothelial cells [2,29,30]. When we cultured THP-1 cells (monocytes) for 24 h, some THP-1 cells formed clumps on the hydrogels (PH and PH/sFDM), whereas TCP showed only single cells. The number of clumps on the PH/sFDM increased with time but TCP showed the increased cell number with single cell morphology (Figure 2D). We postulate that our hydrogel substrate might trigger monocyte activation as a foreign body, rendering them to be clustered. In particular, it is notable that THP-1 cells could release a significantly higher level of VEGF on the PH/sFDM as compared with TCP and PH (Figure 2E). This demonstrates that sFDM in the hydrogel may collectively affect monocytes behavior, such as cell clump formation and paracrine factor (VEGF) release. We assume that the angiogenic factors from sFDM may induce the activation of monocytes as reported elsewhere [31]. The same experiments with macrophages showed no significant difference of VEGF release (data not shown).

To evaluate the therapeutic angiogenesis in vivo, our engineered hydrogel was administered via two different animal models (murine full-thickness wound and hindlimb ischemia). In skin wound models, the PH/sFDM group showed better wound closure and advanced tissue regeneration than the rest of groups (PH and PH/FDM) (Figure 3 and Figure 4). We found that numerous neovessels were developed with the PH/sFDM-treated group in the regenerated tissue, along with the size of newly formed vessels much larger than the others (Figure 4C,D). In this study, our primary interest was to prove therapeutic effects and find any difference between FDM and sFDM. Therefore, we concentrated on three different hydrogel groups (PH, PH/FDM, and PH/sFDM). The defect only group repeatedly showed a poor wound healing in our previous work [26,32]. In addition, PH/sFDM treatment demonstrated enhanced blood reperfusion and a diminishing muscle (gastrocnemius and tibialis) necrosis in the hindlimb ischemic model (Figure 5 and Figure 6). Although the PH group had a lower therapeutic impact than that of PH/sFDM, it also showed a certain level of positive effect on the ischemic wound. In fact, HA is a functional component of connective tissue ECM and plays an important role in tissue development and maintenance of tissue homeostasis [33]. HA is also known to promote angiogenesis by inducing RHAMM-TGFβ [27]. Based on the results of two animal models, we postulate that the angiogenic factors in sFDM may have played a role in triggering angiogenesis in skin wound healing [34] and ischemic injury [35]. More specifically analyzed, DPPIV (CD26) is known as multifunctional glycoprotein that regulates the inflammatory process and accelerates epithelialization in cutaneous wound healing [36]. Coagulation factor Ⅲ (tissue factor) regulates angiogenesis by controlling upregulation of proangiogenic VEGF [37] and uPA promotes angiogenesis by de-repressing VEGF receptor expression [38]. FGF-7 protein binds to its natural receptor FGFR2β that leads to angiogenesis [39] and PTX3 is a major mediator of angiogenesis and neurogenesis after cerebral ischemia and has a significant impact on the recovery of lateral exercise function [40]. Although a distinct or collective mechanism of these factors still remained unclear at this time, we believe that our sFDM acts as a major contributor in tissue regeneration and the exact pathway requires further investigation.

## 4. Materials and Methods

### 4.1. Generation of Fibroblast-Derived Matrix and Decellularization

Human lung fibroblasts (CCL-75; ATCC, Manassas, VA, USA) were cultured in 100 mm culture dish at the density of 2 × 10^4^ cell/cm^2^ in Dulbecco’s modified Eagle’s medium (DMEM) supplemented with 10% fetal bovine serum (FBS), 100 U/mL penicillin, and 100 µg/mL streptomycin under normal culture condition (5% CO_2_, 37 °C). The medium was exchanged every 2–3 days. Those confluent cells were then washed with PBS twice and then subjected to the decellularization process following our protocol as previously reported [32]. In brief, a solution of 0.25% Triton-X 100 and 20 mM NH_4_OH was added to the cells in the plates and those plates were then washed with PBS twice. They were further treated with the solution containing 50 U/mL DNase I and 2.5 μL/mL RNase A for 2 h at 37 °C. After being rinsed with PBS several times, the decellularized FDM was collected in tube using cell scraper and kept at −20 °C with PBS for further use.

### 4.2. Immunofluorescence Staining

The decellularized FDM samples were placed on coverslip and fixed by 4% paraformaldehyde for 30 min at room temperature. After being washed with PBS three times, they were blocked using 3% bovine serum albumin (BSA) for 1 h at room temperature. They were incubated with primary antibody solution overnight at 4 °C, then subsequently rinsed with PBS, added with secondary antibody solution, and incubated for 1 h. Lastly, the stained samples were mounted onto microscope glass slides with vectashield^®^ mounting medium containing 4′, 6-diamidino-2-phenylindole (DAPI) (H1200; Vector Lab, Burlingame, CA, USA). Fluorescence images were captured using confocal laser scanning microscope (LSM 700; Carl Zeiss, Oberkochen, Germany). Mouse anti-fibronectin (SC-8422; Santa Cruz Biotechnology, Dallas, TX, USA) and rabbit polyclonal anti-collagen type I (ab34710; Abcam, Waltham, MA, USA) were used as a primary antibody. Secondary antibodies are Alexa Fluor^®^ 488-conjugated goat anti-mouse IgG (A11001; Invitrogen, Waltham, MA, USA) and Alexa Fluor^®^ 594-conjugated donkey anti-rabbit IgG (A21207; Invitrogen).

### 4.3. Preparation of Suspended FDM Solution and Measurement of Particle Size Distribution

To prepare a suspended FDM (sFDM) dispersion, decellularized FDM was put in DI water or culture media, and pulverized via ultrasonicator (Sonic Dismembrator Model 500, Thermo Fisher Scientific, Waltham, MA, USA). The tube containing the FDM was placed in ice during the sonication to prevent heating and resultant proteins damage. Total proteins in the sFDM were measured via BCA protein assay (23225, Thermo Fisher Scientific, Waltham, MA, USA) according to the manufacturer’s instructions. In addition, to measure the particle size distribution in the homogeneous sFDM dispersion, the sFDM was diluted with PBS at 1:500 ratio. The sample was then transferred into cuvette and examined using Zetasizer Nano (Malvern Panalytical, Malvern, Worcestershire, UK).

### 4.4. Angiogenic-Related Proteins Profiling

To screen angiogenic-related factors in both FDM and sFDM, the Proteome Profiler™ human angiogenesis array kit (ARY007; R&D Systems, Minneapolis, MN, USA) with an array of 55 angiogenesis-related proteins was employed according to the manufacturer’s instructions. Briefly, a nitrocellulose membrane was blocked with a supplied block buffer, then added with a mixture of FDM lysates and a cocktail of biotinylated detection antibodies. Incubated overnight at 4 °C, the membrane was rinsed with a wash buffer, and streptavidin-horseradish and chemiluminescent detection reagents were then added sequentially. Once chemiluminescence was carried out via iBright CL1500 imaging system (Invitrogen, Waltham, MA, USA), the collected data were quantitatively analyzed using iBright analysis software 4.0.1.

### 4.5. Fabrication of sFDM Containing Hydrogel

A paste type hydrogel was fabricated via homogeneously mixing 2 g Pluronic^®^ F127 (P2443, Sigma, Burlington, MA, USA) and 0.15 g hyaluronic acid (HA 1.4; SK Bioland, Osong, Korea) in DI water (7.5 mL) using the ultrasonicator, followed by dilution with DI water (2.5 mL) to produce a Pluronic/HA (PH) hydrogel. The concentration of sFDM dispersion was adjusted to 0.4% (*w/v*) and this sFDM (2.5 mL) was mixed with the concentrated PH hydrogel (7.5 mL) to generate a Pluronic/HA/sFDM (PH/sFDM) hydrogel. To compare the therapeutic efficacy between sFDM and FDM, the FDM was also mixed with the PH hydrogel to make a Pluronic/HA/FDM (PH/FDM). Three different types (PH, PH/FDM, and PH/sFDM) of hydrogels were stored at 4 °C until further use.

### 4.6. Characterization of Hydrogel: Scanning Electron Microscope and Rheology

For the analysis of a microstructure of sFDM or FDM containing hydrogels, they were kept at −80 °C and lyophilized. The freeze-dried samples were placed on the aluminum stubs, then platinum-coated for 1 min and, observed using scanning electron microscope (SEM, Phenom G2 pro desktop; Eindhoven, Netherlands). Meanwhile, some rheological properties of the hydrogels were also determined using Anton Paar Rheometer (MCR102; Anton Paar, Graz, Austria). A parallel plate of 25 mm diameter was prepared with the sample gap size of 0.3 mm. Both storage (G’) and loss modulus (G”) were measured while applying 2% of shear strain under specific temperature and frequency ranges.

### 4.7. Examination of HUVEC Response Using sFDM

The sFDM added medium was prepared in endothelial cell basal medium (EBM-2, CC-3156; without supplements kit), which was diluted to the final concentration of 0.01 and 0.1 mg/mL sFDM, respectively. EBM-2 was used as a serum-free medium. Human umbilical vein endothelial cells (HUVEC, C2517A; Lonza, Basel, Switzerland) were cultured in 12-well plates for 24 h with endothelial cell growth medium (EGM-2 BulletKit, CC-3162; EBM-2 with supplements kit). Once the EGM-2 medium was removed, three different kinds of medium (EBM-2, EBM-2 with 0.01 mg/mL sFDM, and EBM-2 with 0.1 mg/mL sFDM) were separately replenished to each well. The cell morphology was observed using optical microscope. The cell proliferation was also examined at 24 and 72 h (*n* = 3, each group) using cell counting kit-8 (CCK-8) assay (CK04; Dojindo, Kumamoto, Japan).

### 4.8. Monocyte Culture on PH/sFDM Hydrogel and Measurement of VEGF Release

To assess an immune response of the hydrogels, we prepared three different groups in 24-well culture plates (*n* = 4, each group): tissue culture plate (TCP), PH hydrogel-coated TCP, and PH/sFDM hydrogel-coated TCP. The coating volume of each hydrogel was 100 µL per well and the hydrogel-coated plates were kept at 37 °C for 3 min to induce gelation. Human peripheral blood monocytes (THP-1, TIB^®^-202^TM^; ATCC, Manassas, VA, USA) were seeded in the prepared culture plates (TCP, PH, and PH/sFDM) at the density of 3 × 10^5^ cell/mL and cultivated for 3 days with Roswell Park Memorial Institute (RPMI) media (Gibco, Waltham, MA, USA), supplemented with 10% FBS, 100 U/mL penicillin, and 100 µg/mL streptomycin under normal culture condition. To measure the amount of VEGF released from THP-1 grown on different substrates, each culture medium was centrifuged and the supernatant was collected, followed by the measurement of VEGF concentration using VEGF ELISA kit (DVE00; R&D system).

### 4.9. Evaluation of Therapeutic Effect of Hydrogel Using Full-Thickness Wound Model

All the animal studies were performed in accordance with the Korea Institute of Science and Technology Animal Care and Use Committee Guidelines (KIST-2019-017). The BALB/c nude mice (male, 6 weeks old) (Orient Bio, Gapyeong, Korea) were randomly divided into three groups: PH, PH/FDM, and PH/sFDM (*n* = 3 per group/2 wounds per mouse). They were anesthetized by gas inhalation using isoflurane in oxygen prior to surgery and the dorsal skin was then scrubbed using alcohol gauze. Full-thickness wounds were created using a biopsy punch (6 mm) under sterile surgical condition. Those hydrogels were kept at 37 °C for gelation before transplantation. They were subsequently injected through a syringe needle and pasted over the wounded area, then Tegaderm™ Film and coban bandage were wrapped around the wounds to protect the treatments. The wound closure was grossly observed on day 0, 3, 7, 10, and 14 post surgery while the hydrogels were replaced every 3–4 days. The wound area at specific time points (four random images, each group) was quantitatively calculated using ImageJ software as a percentage of the skin wound area normalized to that of day 0. Three different concentrations (0.01, 0.1, and 1%) of sFDM hydrogel groups (*n* = 2 per group/2 wounds per mouse) was used for optimizing sFDM concentration. This preliminary test was also performed similarly with the same process above.

### 4.10. Histological Analysis of the Regenerated Tissue in the Wounded Area

The skin wound tissues were excised after those mice were euthanized using CO_2_ inhalation on day 14. The samples were fixed with 10% formalin at room temperature, embedded in paraffin block, then sectioned in 6 μm thickness across the tissues. These sections were deparaffinized using xylene and rehydrated in a series of alcohol solutions. To evaluate wound healing in the wounded area, they were subjected to H&E and Herovici staining (KTHERPT; StatLab, Mckinney, TX, USA), respectively. Once the stained images were taken using inverted microscope (Axio Vert.A1, Carl Zeiss, Oberkochen, Germany), epidermal thickness, neovessel size, and mature collagen deposition were quantitatively determined using ImageJ software 1.52a (four random images, each group).

### 4.11. Evaluation of Therapeutic Effect of Hydrogel Using Hindlimb Ischemia Model

Male BALB/c mice (8 weeks old) were selected and surgically operated to create hindlimb ischemia after being anesthetized with Zoletil (30 mg/kg) and rumpun (20 mg/kg). All the animal studies were approved by the Institutional Animal Care and Use Committee (IACUC) (approval number, 2018-0185), Yonsei University College of Medicine. The left leg was then ligated with 7.0 prolene from the profunda femoral artery to the distal popliteal artery, as well as to the superficial femoral artery and epigastric artery. Eventually the entire femoral artery was excised. Administration of PBS, PH, and PH/sFDM was conducted in the ischemic regions (six animals per group). The amount of administered hydrogel was 0.15 g at a time. Subsequently, the skin incision was closed using 7.0 prolene. Those animals were taken care of for 3 weeks before sacrifice.

### 4.12. Laser Doppler Perfusion Imaging (LDPI)

Blood perfusion of hindlimb was monitored via a laser Doppler perfusion image (Moor Instruments, Devon, UK) on pre- and post-operative day 0, 7, 14, and 21. To compensate for variations in light and temperature during imaging, changes in blood perfusion were calculated using region-based thresholding as the ratio of perfusion in the ischemic limb (left) to the perfusion in the normal limb (right).

### 4.13. Histological Analysis of Ischemic Hindlimb

The gastrocnemius muscle and tibialis muscle tissues were excised from the euthanized mice using CO_2_ inhalation on day 21 and fixed with 10% formalin at room temperature. Once embedded in paraffin block and sectioned in 5 µm thickness, those sections were deparaffinized, rehydrated, then subjected to H&E and MT staining. All the stained sections were photographed using microscope (Olympus BX53, Tokyo, Japan) and the software (cellSens, Zuid-Holland, Netherlands). Signs of inflammation, degeneration, and fibrosis within the hindlimb muscles were examined using H&E and MT images, respectively and quantified using ImageJ software. We took three randomly chosen fields out of 5~7 different tissue sections per animal for quantitative analysis.

### 4.14. Statistical Analysis

All data presented are mean ± standard deviation. Statistical analyses were performed using one-way analysis of variance (ANOVA) with a post-hoc Tukey’s multiple comparison test via GraphPad Prism 9 software. A statistically significant difference is denoted as * *p* < 0.05, **p < 0.01, ***p < 0.001, or ****p < 0.0001.

## 5. Conclusions

This study demonstrates the therapeutic effect of sFDM via a deep skin wound and hindlimb ischemia model in vivo. We have successfully developed a paste type hydrogel formulation by combining sFDM with Pluronic F127 and HA hydrogel. sFDM holds nano-sized ECM particles and contains various bioactive molecules. Our PH/sFDM hydrogel is biocompatible, mechanically stable, injectable and can be easily administered in the target regions. It was notable that sFDM can enhance cell survival and trigger VEGF secretion from immune cells. Moreover, our PH/sFDM hydrogel presented advanced therapeutic effects via a deep wound model in vivo, as proven by the epidermal thickness similar to normal skin, better neovessels formation, and improved mature collagen deposition. The hindlimb ischemia model also showed therapeutic improvement with PH/sFDM hydrogel treatment. In this study, we confirm that sFDM itself possesses a strong therapeutic potential and thus our engineered hydrogel (PH/sFDM) should be a promising material in the area of tissue engineering and regenerative medicine.

## Data Availability

Not applicable.

## Supplementary Materials

The following are available online at https://www.mdpi.com/article/10.3390/ijms222413265/s1.
