# Supplementary material for: Nano-Sized Extracellular Matrix Particles Lead to Therapeutic Improvement for Cutaneous Wound and Hindlimb Ischemia
Source: Int J Mol Sci. 2021 Dec 9;22(24):13265. doi: 10.3390/ijms222413265 (PMC8705579; doi:10.3390/ijms222413265)
Supplement: Supplementary file 1 [file ijms-22-13265-s001.zip › ijms-1460724-supplementary.pdf]

SUPPORTING INFORMATION

Nano-sized Extracellular Matrix Particles Lead to Therapeutic Improvement for Cutaneous Wound and Hindlimb Ischemia

Sang Su Ha [1,†], Jung-Hyun Kim [3,†], Cininta Savitri [1,2], Donghoon Choi [3,*],and Kwideok Park [1,2,*]

[1] Center for Biomaterials, Korea Institute of Science and Technology (KIST), Seoul 02792, Republic of Korea, 024730@kist.re.kr (S.S.H.); casavitri@kist.re.kr (C.S.)

[2] Division of Bio-Medical Science and Technology, KIST School, University of Science and Technology (UST), Seoul 02792, Republic of Korea

[3] Division of Cardiology Department of Internal Medicine, Severance Cardiovascular Hospital Yonsei University College of Medicine, Seoul 03722, Republic of Korea, JHKIM915@yuhs.ac (J.H.K.)

[*] Correspondence: CDHLYJ@yuhs.ac (D.C.); kpark@kist.re.kr (K.P.)

[†] These authors contributed equally to this work.

Running title: Nano-sized extracellular matrix particles for therapeutic improvement

Submitted to *International Journal of Molecular Sciences*

November 2021

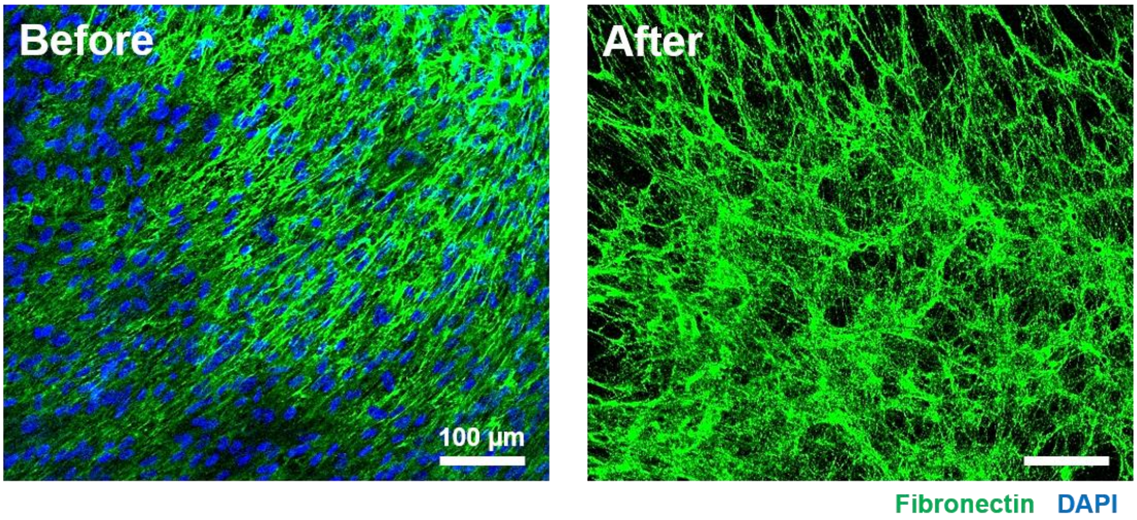

Figure S1. Preparation of FDM via decellularization. The representative images of human lung fibroblasts before and after decellularization via immunofluorescence: fibronectin (green) and 4', 6-diamidino-2-phenylindole (DAPI, blue).

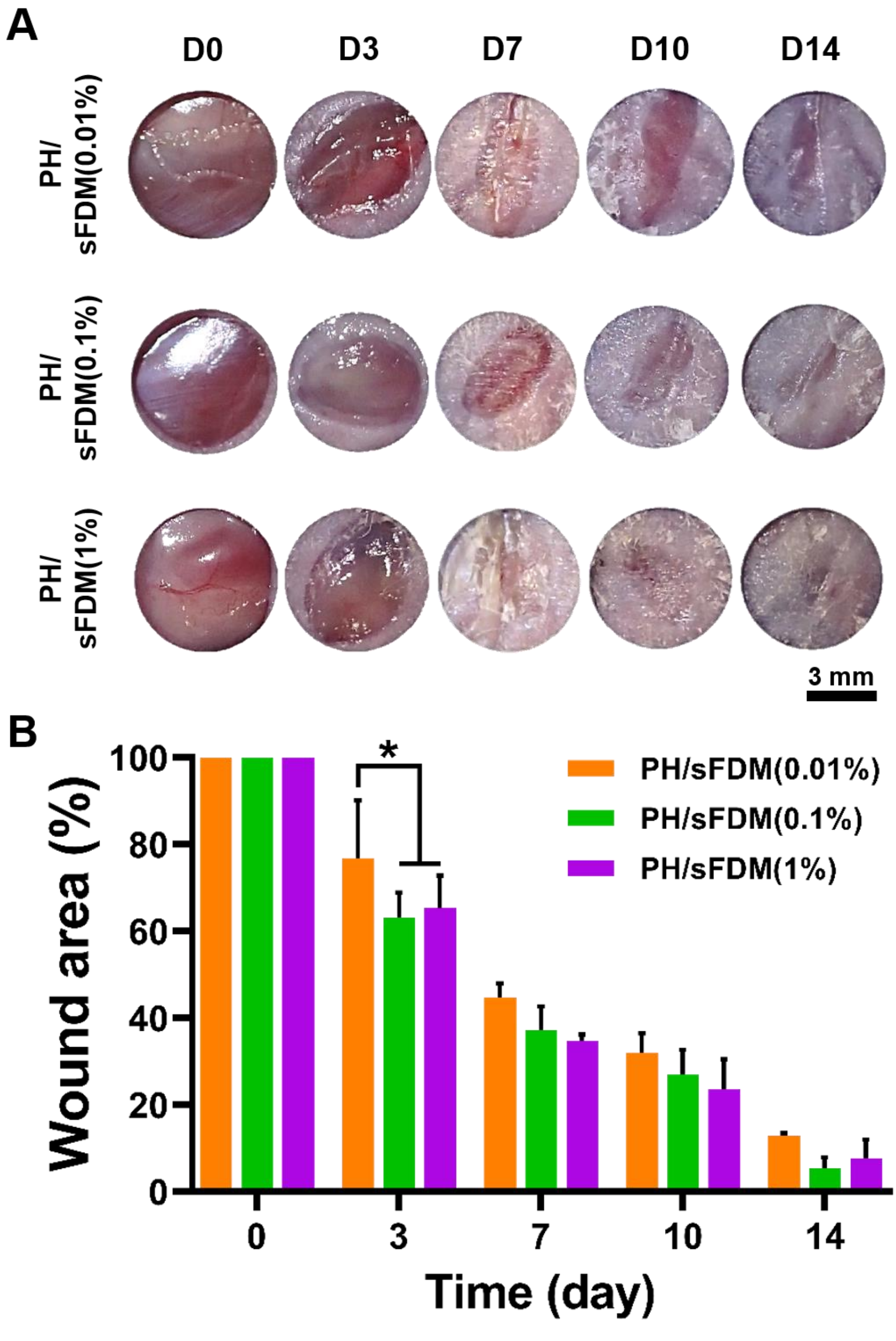

Figure S2. Skin wound closure test for optimizing sFDM concentration. (A) Representative images of wound closure using three hydrogels with different concentrations (0.01, 0.1, and 1%, respectively) of sFDM on day 3, 7, 10, and 14 post-surgery. (B) Measurement of wound area size (%) at specific time points for up to 14 days.
